# Simulated microgravity in the ring-sheared drop

**DOI:** 10.1038/s41526-019-0092-1

**Published:** 2020-01-03

**Authors:** Patrick M. McMackin, Shannon R. Griffin, Frank P. Riley, Shreyash Gulati, Nicholas E. Debono, Aditya Raghunandan, Juan M. Lopez, Amir H. Hirsa

**Affiliations:** 1 Mechanical, Aerospace, and Nuclear Engineering, Rensselaer Polytechnic Institute, Troy, NY 12180-3590 USA; 2 School of Mathematical and Statistical Sciences, Arizona State University, Tempe, AZ 85287 USA; 3 Department of Mechanical, Aerospace and Nuclear Engineering (also, Chemical and Biological Engineering), Rensselaer Polytechnic Institute, Troy, NY 12180-3590 USA

**Keywords:** Fluid dynamics, Biophysics

## Abstract

The ring-sheared drop is a module for the International Space Station to study sheared fluid interfaces and their influence on amyloid fibril formation. A 2.54-cm diameter drop is constrained by a stationary sharp-edged ring at some latitude and sheared by the rotation of another ring in the other hemisphere. Shearing motion is conveyed primarily by the action of surface shear viscosity. Here, we simulate microgravity in the laboratory using a density-matched liquid surrounding the drop. Upon shearing, the drop’s deformation away from spherical is found to be a result of viscous and inertial forces balanced against the capillary force. We also present evidence that the deformation increases with increasing surface shear viscosity.

## Introduction

**Fig. 1:**
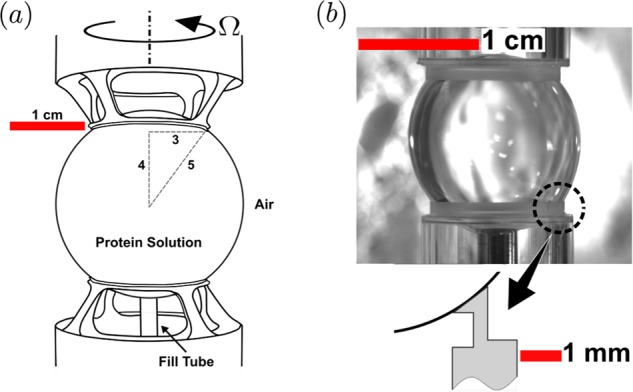
Overview of hardware. (**a**) Schematic of the Ring-Sheared Drop (RSD) hardware onboard the ISS, built by Teledyne–Brown Engr. Corp. (**b**) Image of a protein drop solution (insulin) pinned to polycarbonate contact rings taken during a parabolic flight (November 2017).

The prospects of containerless material processing in microgravity has spurred on several decades of productive research. This research culminated in numerous experiments in low-Earth orbit, some of which are ongoing.^1–4^ Aside from removing complications associated with solid containers, the absence of natural convection afforded by microgravity has been essential for much of the materials research, such as growing 3D crystals under quiescent or very slow and controlled flow conditions.^5–7^ On the other hand, in bioreactor applications as well as in many biophysics studies, some agitation, or imposed shear flow is essential.^8^ In a new module for the International Space Station (ISS), the Ring-Sheared Drop (RSD, see Fig. 1a), surface tension provides containment at the centimeter scale, as in many other microgravity experiments. However, the RSD utilizes another interfacial property to convey flow, namely surface shear viscosity. A significant shearing motion throughout the drop is produced by merely rotating a contact ring along the surface of the drop. In the presence of macromolecules, such as proteins or microorganisms and their byproducts, the air-water interface exhibits sufficient surface shear viscosity to enable strong bulk flow in drops at scales of up to several centimeters.^9^

The RSD module was designed to impart shear on a dilute protein solution and study the resulting amyloid fibrils that form at the fluid interface and in the bulk, while minimizing contact with solid surfaces. Amyloid fibril formation is of wide interest due to the central role that amyloid protein structures play in many neurodegenerative disorders, including Alzheimer’s and Parkinson’s diseases. The RSD experiment consists of a 2.54-cm diameter drop of dissolved protein (insulin in the present ISS experiments) that is constrained by two thin contact rings; see Fig. 1a. The surface area of the drop that is in contact with the rings in the present space hardware is ~5% of the total surface area of the drop. The finite thickness of the contact rings (~1.2 mm) is due to practical considerations, namely the manufacturing of rings with sharp edges to ensure proper pinning of the drop, and not necessarily for the shearing flow. It has been established in a closely related flow apparatus (the knife-edge surface viscometer) that the contact ring thickness plays a secondary role in the hydrodynamics.^10^

A few studies on the RSD have appeared in the literature. The formation of the drop and its pinning by two contact rings was reported in Gulati et al.^11^ They used a combination of laboratory experiments, numerical simulations, and parabolic flights to study water drop formation and pinning in the RSD geometry, using drops of 1.0 cm diameter. A second parabolic flight campaign successfully demonstrated the growth and pinning of drops of 1.5 cm diameter; see Fig. 1b which includes an inset showing the design of the contact rings in that flight. However, the duration of microgravity in parabolic flights is barely long enough to grow and pin a drop the size of the RSD for ISS, and orders of magnitude shorter than the hours or days needed for studying shear-induced fibrillization in the RSD module aboard the ISS.

Two subsequent studies, both computational and without gravity, have reported on the flow in a spherical drop that is sheared by sharp-edged contact rings. Specifically, Gulati et al.^12^ presented numerical simulations of the flow in the RSD and its implications on mixing within the drop. For that study, the surface shear viscosity ${\mu }^{s}$ was assumed to be large compared to the product of the viscosity in the bulk, $\mu$, and the drop radius, $R$. This ratio is the Boussinesq number $Bo\ =\ {\mu }^{s}/\mu R$. In the limit $Bo\ \to \ \infty$, the interfacial flow decouples from the bulk flow, allowing for an analytic solution for the velocity on the surface of the drop. The analytic solution was then used as the boundary condition for the bulk flow simulations. Although the flow on the surface of the drop is decoupled from the bulk flow in this limit, the flow inside the drop depends on the surface flow. More recently, this restriction was removed by considering a large range in $Bo$, and the coupled flow in the RSD was studied numerically with arbitrarily small surface shear viscosity.^9^

Here, we present the first experimental results on the flow in an RSD, where a full-scale drop (nominal diameter of 2.6 cm) is constrained by a stationary contact ring and sheared by the constant rotation of a second ring. The objective of the experiment is to demonstrate the robustness of the RSD flow system, showing the range of parameters where the drop is nominally spherical and where drop deformations become significant. Microgravity is simulated in the laboratory using a density-matched liquid surrounding the drop. The combination of the drop fluid and surrounding fluid was selected with a large viscosity ratio to minimize friction effects of the surrounding fluid in order to approximate the flow of an aqueous drop surrounded by air in the ISS. The experimental results are presented along with numerical simulations of idealized spherical drops.

## Results

### Experimental apparatus and procedures

**Fig. 2:**
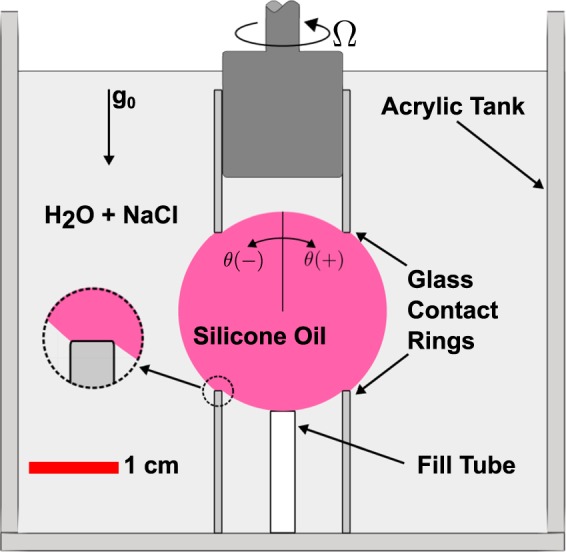
Schematic of the RSD experiment simulating microgravity on Earth. Note, the oil drop wets the end of each glass tube, forming contact rings.

The experiments were performed in a 7.5-cm tall, 6.0 cm × 6.5 cm acrylic open test cell. Droplets of silicone oil were grown from the tip of a stainless steel fill tube (0.33 cm outer diameter and 0.26 cm inner diameter), mounted on the bottom of the container, see Fig. 2. Contact rings were formed at one end of glass tubes that were cut from standard test tubes ($1.582\pm 0.004$ cm outer diameter and $0.081\pm 0.001$ cm wall thickness). The ends of the tubes were ground flat using a procedure described in Gulati et al.^11^ Glass was selected as the contact ring material for these experiments due to its large contact angle with silicone oil immersed in water. The static contact angle of sessile oil drops was measured independently on clean glass slides in water and found to be larger than what is required from the geometry of the drop and contact ring ($\approx\!\!12{7}^{\circ }$). This prevented the oil from depinning from the rim and wetting the inside of the glass tubes.

One ring was located at the bottom of the container, centered on the fill tube to within 0.002 cm. The other ring was fitted on a stepper motor placed atop of the test cell. The largest geometric imperfection in the system was the lateral motion of the rotating ring. The wobble was $\pm$0.03 cm. A three-axis-micrometer stage lowered the top ring into the container, enabling the rings to be aligned and placed 1.83 cm apart. This resulted in a 3:4:5 right-triangle geometry between the edge of the ring and the center point, thus matching the RSD geometry; see Fig. 1a.

Two types of silicone oil were used in experiments: a high viscosity oil (Gelest, PDMS-0821) and a low viscosity oil (Gelest, PDMS 7040). The high viscosity silicone oil was also used with a dye at a concentration of 1 mg of DilC${}_{18}$ (Invitrogen, D282) per mL of PDMS (0821). The nominal kinematic viscosity of the high viscosity oil was measured and found to be $121.4\pm 1.0$ cSt, within the range given by the manufacturer of 100–125 cSt. Note that all the measurements and experiments were conducted at room temperature ($23.5\pm 0.{5}$ °C). The viscosity of the low viscosity oil was measured to be $37.1\pm 0.3$ cSt, which is also within the manufacturer’s range of 35–40 cSt. The viscosity of the dyed oil was measured to be $108.5\pm 1.0$ cSt. All three types of oil (high viscosity, low viscosity, and high viscosity with dye) were found to be heavier than water even though the low viscosity oil was specified by the manufacturer to be lighter than water. The specific gravity (i.e. density at room temperature divided by density of water at 4 °C) of the high viscosity oil, low viscosity oil, and high viscosity oil with dye was measured to be, respectively, $1.002\pm 0.001$, $1.037\pm 0.025$, and $0.996\pm 0.007$.

The test cell was first filled with a NaCl/De-ionized (DI) water solution. The concentration of NaCl was varied subsequently to density match the silicone oil used to grow the drop, simulating microgravity. Fine tuning of the density-matching by adjusting the molarity of the saline solution was accomplished by visually judging the the sphericity of the drops on the camera. The final molarity of the saline solution was 1.55 M for the low viscosity oil, 0.14 M for the high viscosity oil, and 0.13 M for the dyed high viscosity oil. The measured specific gravity of the silicone oils agreed with their corresponding saline solution specific gravity,^13^ to within experimental uncertainty.

The interfacial tension of each of the three oils against a saline solution was measured and found to be $32.4\pm 1.6$, $38.1\pm 1.5$, and $39.4\pm 1.4$ dyn/cm for the high viscosity oil, low viscosity oil, and high viscosity oil with dye, respectively. These values were in the range reported in the literature for silicone oil against water.^14–16^

Before each experiment, the glass contact rings were cleaned with methylene chloride, then with reagent grade acetone, rinsed with DI water, and then rinsed with acetone. The test cell was cleaned using RBS detergent, then rinsed with DI water, and finally wiped with acetone. The drops were imaged with a Nikon D5500 camera, using a Micro-NIKKOR 105-mm focal length lens.

**Fig. 3:**
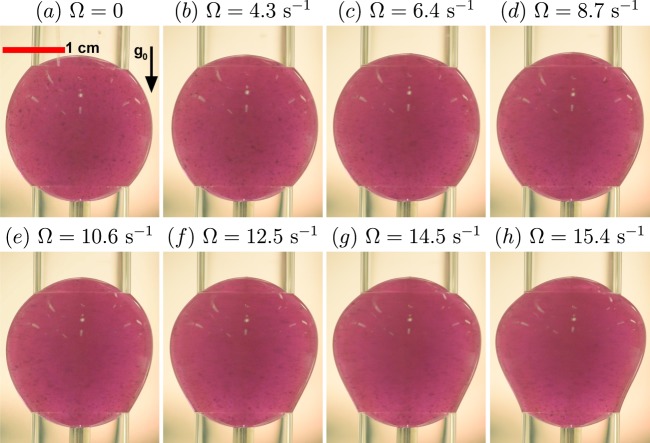
Snap-shots from videos of the drop of high viscosity silicone oil with dye at various top-ring rotation rates $\Omega$, as indicated.

In order to study drop deformation, the experiment was run at the desired speed, stopped, and then run at the next speed. The results presented in Fig. 3, described in the next section, were obtained by running the ring for at least 6 s and taking a video, with conventional lighting, at 30 fps for 4 s for a total of 120 frames. It should be noted that the viscous time, ${R}^{2}/\nu$ where $\nu$ is the kinematic viscosity, for the high viscosity oil drops is ~1 s and the viscous time for the low viscosity oil drop is ~4 s. The measurements for the high viscosity oils were initiated after at least 2–4 s and the measurements for the low viscosity cases were commenced after 5–6 s. In either case, there was no detectable change in the drop shape for the 4-s duration of the measurements, indicating that the flows had nominally reached steady state.

To collect azimuthal velocity data, images and videos were captured with the drop illuminated by a laser light sheet generated by a 10-mW 450-nm laser. A density-matched saline solution with fluorescein added at a concentration of 0.5 ppm filled the test cell. High viscosity oil dyed with partially dissolved DilC${}_{18}$ (as described earlier) was used to grow the drop, and particles of undissolved dye near the drop surface were tracked.

### Experimental results

Experiments were conducted with silicone oils of two different viscosities. The more viscous of the two oils was also used with a dye added to it, providing three different drop fluids. Originally, the oil-soluble fluorescent dye was added to enhance contrast and to aid visualization. The dye was found to only partially dissolve, forming small domains which could be tracked, thus enabling velocimetry. Fortuitously, the dyed oil drops were found to have noticeably different shape at a given rotation rate than the same oil without the added dye, thus providing useful data and insights, as will be shown later.

Snapshots of the high-viscosity oil drop with dye are presented in Fig. 3 for various top-ring angular velocities $\Omega$. The static case (Fig. 3a) shows an essentially spherical drop, except at the rim of the glass tubes which appear to be wetted, where contact ring has a thickness equal to the glass tube wall thickness (0.8 mm). The contact patch is comparable to the RSD space hardware (see Fig. 1a), except that the latter is contoured to the shape of the drop whereas the rim of the glass tubes used in the present experiments are cut square (see Fig. 2).

**Fig. 4:**
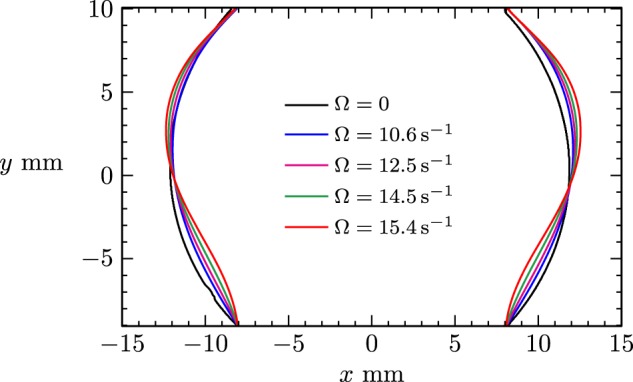
Location (relative to the center of the RSD) of the drop edge, time-averaged over 120 frames (4 s duration) of video images of the type shown in Fig. 3, for various $\Omega$ as indicated.

Figure 3 shows that there is little deviation in the drop shape with increasing ring rotation speed for $\Omega\, \lesssim \ 10.6\;{{\rm{s}}}^{-1}$. Note that the maximum speed for the present oil–water system experiments, $\Omega =29\ {{\rm{s}}}^{-1}\approx \ 275\ {\rm{rpm}}$ obtained with the low viscosity oil, is an order of magnitude larger than the anticipated maximum operating speed of the RSD module on the ISS, estimated to be $\Omega =\pi {s}^{-1}=\ 30\ {\rm{rpm}}$.^9^ However, it should be noted that deformation in a rotating drop is expected to be a function of density difference between the drop and the surrounding fluid.^17,18^ Figure 3 also shows that at faster speeds, the drop widens near the rotating ring and narrows near the stationary ring. The drop deformations for various $\Omega$ were extracted from images like those in Fig. 3 and the time-averaged drop radii are plotted in Fig. 4. The figure shows that the drop bulges radially outward on the side of the rotating ring (i.e. in the northern hemisphere) and is drawn radially inward on the stationary ring side. These distortions are not left-right symmetric, and videos of the flow are suggestive of the presence of a helical wave mode. If only inertial effects were important, one might expect the drop to spin as a solid-body at $0.5\,\Omega$ as suggested by Gulati et al.^9^ and bulge out at the equator. However, as we will show below, viscous effects appear to play a significant role here.

**Fig. 5:**
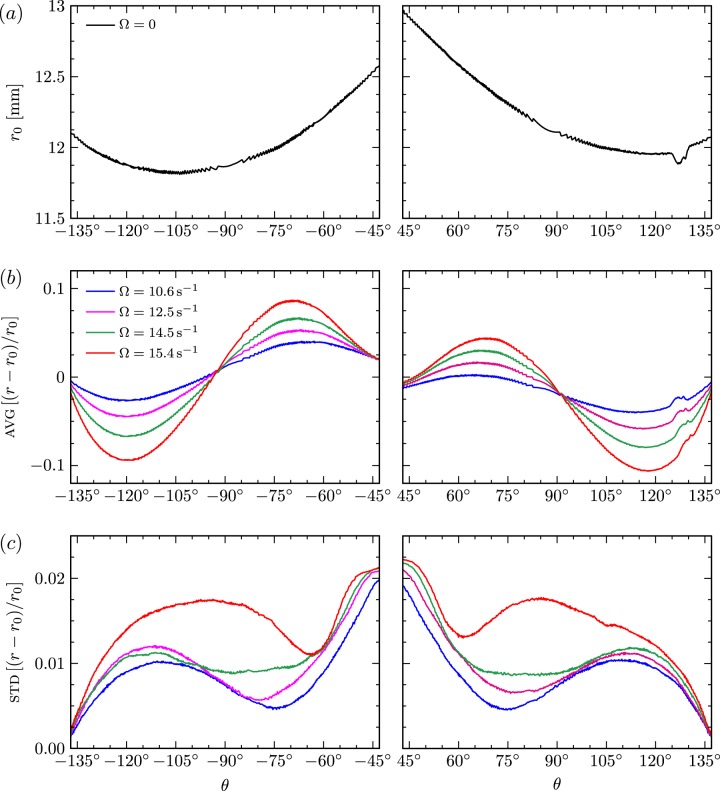
Expirimental data. (**a**) Drop radius as a function of polar angle of the static drop ($\Omega =0$) of high viscosity oil with dye. The left and right time-averages AVG (**b**) and standard deviations STD (**c**) of the drop radius deformations relative to the radius of the static drop for various $\Omega$, as indicated. The polar angles range over $\theta \in [\pm {39}^{\circ },\pm {137}^{\circ }]$, from the rotating ring at $\theta =\pm {39}^{\circ }$ to the stationary ring at $\theta \pm {137}^{\circ }$.

In order to quantify the effects of ring rotation on drop shape, first the radius of the static drop ($\Omega =0$) was examined. Figure 5 shows the radius of the static drop, ${r}_{0}(\theta )$, from the geometric center, for polar angles $\theta$ between the stationary and rotating rings; negative (positive) $\theta$ corresponds to the left (right) part of the drop surface in images like those in Fig. 4, with $\theta ={0}^{\circ }$ at the north pole (top of the image). The geometric center was determined from the intersection of the line connecting the top right point of pinning (of the drop and glass ring) to the bottom left point of pinning and the line connecting the top left to the bottom right points of pinning. This allowed for the radius as a function of $\theta$ to be readily measured for both sides of the image of the drop. Note that the data in Fig. 5 is unfiltered and that the irregularities in the drop shape around $\theta =12{8}^{\circ }$ are an optical effect caused by light reflection from the drop, resulting in inaccuracies in the automated edge detection.

The polar deviation of the static drop radius from a constant value is a result of three distinct effects. First, if the drop is over-filled or under-filled for the given ring size and spacing, then the center of curvature moves from the origin to a different location. Second, if the density of the surrounding aqueous solution is not matched perfectly to the drop density, then the drop is deformed due to gravity, tending to either float or sink. In the case presented in the figure, the drop appears to be slightly buoyant and thus tending to rise. The third reason for the radial deviations is the imperfect wetting of the rings, as well as other geometric imperfections in the flow apparatus. The total deviation in ${r}_{0}$ due to the combined effects was kept below 10% for all the experiments.

Figure 5 also shows the time average, AVG, and standard deviation, STD, of the deformations relative to the shape of the static drop. For rotation speeds $\Omega\, \lesssim \ 10.6\ {{\rm{s}}}^{-1}$, there was little departure in drop shape from its static shape, and the corresponding curves fall nearly on top of each other. Figure 5 shows monotonically increasing departure in radius with increasing $\Omega$. Aside from these asymmetries, overall, the time-averaged drop shape, as well as the standard deviation, show left-right asymmetry, suggesting that the instabilities in the drop shape up to the maximum speed presented ($\Omega =\ 15.4\ {{\rm{s}}}^{-1}$) is a rotating helical instability. Some of this asymmetry is present for small and zero $\Omega$, but between $\Omega =\ 14.5\ {{\rm{s}}}^{-1}$ and $\Omega =\ 15.4\ {{\rm{s}}}^{-1}$ there is a large increase in std in an equatorial bulge (around $\theta =\pm 9{0}^{\circ }$), indicative of an imperfect symmetry-breaking bifurcation at which the helical wave becomes predominant. This level of $\Omega$ correlates with flow inertia becoming important. The Reynolds number $Re\ =\ \Omega {R}^{2}/\nu$ (ratio of inertial and viscous stresses) for this transition is $Re\ =\ 23.5\pm 0.5$.

A global measure of the drop deformation for each angular speed of the ring, $\Omega$, was determined as follows. First, each radius from the video images for $\Omega \,\ne\, 0$ was time averaged over 4 s (120 frames) once the system had reached steady state. The local, time-averaged radius was then averaged between left (negative $\theta$) and right (positive $\theta$) sides of the drop image for each rotation rate and defined as ${\widehat{r}}_{\Omega }(\theta )$. These were then normalized relative to the $\Omega =0$ case and integrated over $\theta$, to get a global measure of the deformation

1 $${\widehat{R}}_{\Omega }={\int }_{{\!39}^{\circ }}^{{137}^{\circ }}| {\widehat{r}}_{\Omega }(\theta )-{r}_{0}(\theta )| /{r}_{0}(\theta )\ {\rm{d}}\theta \ .$$

**Fig. 6:**
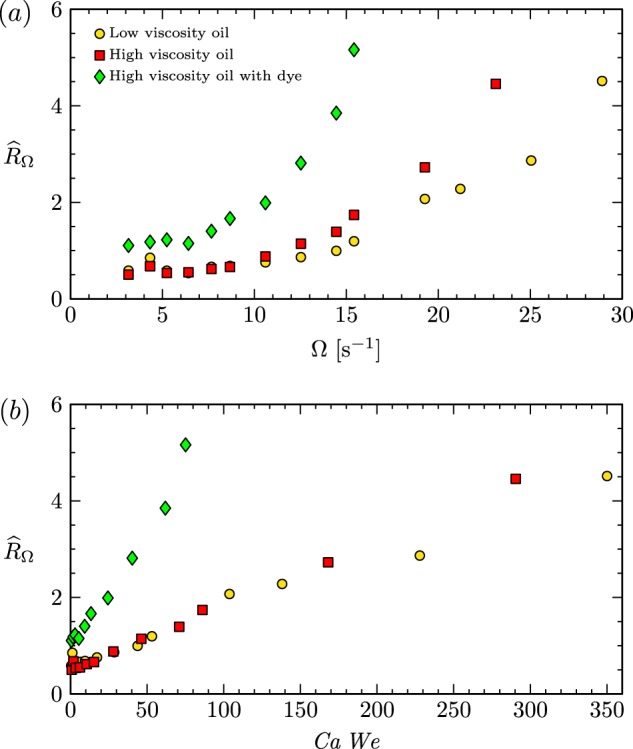
Resulting plots from expirimental data. (**a**) The drops’ relative mean deformation, integrated between the stationary and rotating ring, as a function of $\Omega$ for each of type of silicone oil. (**b**) The same data as in **a**, but recasting $\Omega$ in non-dimensional terms as the product of the capillary and Weber numbers $Ca\ We$.

The results, presented in Fig. 6a, show disparate responses for the three different oils, with the largest deformation in the high viscosity oil with dye case and the smallest deformation in the low viscosity oil case. The results delineate the range of ring angular speed where the RSD has minimal deviation from spherical in each case. It should be noted that wetting and geometric imperfections in the experiment are the primary reasons for the difference in the baseline value in the case of high viscosity oil with dye versus the other two cases (low viscosity and high viscosity with no dye). A major contributor to the error in the ${\widehat{R}}_{\Omega }$ is the azimuthal position where the rotating ring happens to be for determining ${r}_{0}(\theta )$ due to the ring wobble, described above.

In all three cases, there is little change in ${\widehat{R}}_{\Omega }$ for the first four ring speeds (i.e. up to $\Omega =\ 6.4\ {{\rm{s}}}^{-1}$). The change in ${\widehat{R}}_{\Omega }$ grows rapidly with increasing $\Omega$, and the responses for the three different oils diverge at large $\Omega$.

Empirically, we found that plotting ${\widehat{R}}_{\Omega }$ against the product of the capillary number $Ca\ =\ \nu \rho \Omega R/\sigma$ (ratio of viscous and capillary stresses) and the Weber number $We\ =\ \rho {\Omega }^{2}{R}^{3}/\sigma$ (ratio of inertial and capillary stresses) collapses the data for the high and low viscosity oil. This suggests that both viscosity and inertia are important in balancing the capillary force in the ring-sheared drop. Figure 6b presents the total deformation as a function of $Ca\ We$ showing that the two oils that were not dyed behave similarly, with a response that is very different from the dyed case. Computations with spherical drops have shown that flow in the azimuthal plane (secondary flow) increases significantly in the presence of surface shear viscosity and the observed drop shape is consistent with the secondary motion induced by surface shear viscosity.^9^ In other words, we believe that an additional dimensionless group, namely Boussinesq number $Bo$, also governs the drop deformation. A large $Bo$ drives a strong meridional flow,^9^ which we believe increases the overall drop deformation. The surface of the oils without dye appear to be clean and thus exhibit low surface shear viscosity and low $Bo$ which leads to less deformation.

**Fig. 7:**
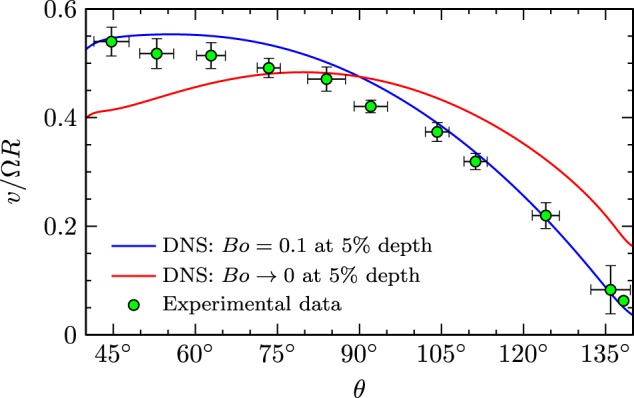
The azimuthal velocity measured near the interface of the high viscosity silicone oil with dye drop, compared to computational results with *B*o = 0.1 at a depth of 5% below the drop interface and Bo $\to$ 0. For presentation purposes, the experimental measurements were averaged over approximately ${10}^{\circ }$ increments with error bars showing one standard deviation.

To better understand the dynamics of the ring-sheared drop, azimuthal velocity measurements were made as close to the drop surface as possible. The measured velocity profiles, scaled by the angular velocity of the ring and drop radius, are presented in Fig. 7. The measurement was done for the case with ring speed set to the maximum expected value for the ISS experiment, namely $\Omega =\pi {s}^{-1}=\ 30\ {\rm{rpm}}$. The measured azimuthal velocity profile was found to correlate best to the computed velocity not for an inviscid surface ($Bo\ \to \ 0$), but one with surface shear viscosity corresponding to $Bo\ =\ 0.1$. This is consistent with the observation that this drop fluid (high viscosity with dye) exhibits the greatest deformation at large $\Omega$, presumably as a result of greater secondary flow. Note, since the domains that were tracked to determine the velocity profile were not always exactly at the surface, we compare with the computed velocity profile at a depth of 5%, i.e. for $r=0.95R$.

## Discussion

The experiment presented here is not a microgravity experiment, but it does explore some of the hydrodynamic issues that are associated with the ISS experiment that it tries to model. Of course, using a density-matched oil-water system to reduce gravitational effects on Earth works quite well, but at the same time the interfacial properties are quite different from those of an air–water system. The ISS experiments will have protein and fibrils on the interface, and how these affect drop deformation, favorably or otherwise, remains to be seen. The behavior of protein at oil–water interfaces is different to that of air–water. Parabolic flight experiments are of too short duration and the present Earth-based RSD experiment cannot address this. Nevertheless, the present experiments using a version of the RSD that is much less precise than the space hardware, driven at an order of magnitude faster rotation speeds, show that the drops, although deformed away from spherical by up to 10% in radius, hold together well.

Finally, it should be emphasized that the deformations associated with a liquid drop in air, corresponding to the RSD on the ISS is fundamentally different from the deformation studied here. Shape and stability of rotating drops in fluids with a finite density difference has been studied by refs. ^17,18^ among others. In that problem, the deformation diminishes if there is no density difference between the drop and its surrounding fluid. The situation in the RSD is different as we expect deformation due to the differential rotation at different latitudes and secondary flow even if completely density matched.

## Methods

### Fluid property measurements

Densities were determined by measuring volume with either 25 mL or 10 mL precision volumetric flasks, and mass using an analytical balance with an uncertainty of 0.04 mg. Viscosities of the two Gelest oils and the dyed high viscosity oil were measured with a rheometer (Anton Paar MCR 301), using a cone and plate configuration.

Interfacial tension ($\sigma$) was measured using the pendant drop method.^19^ For the drop to form the pendant shape analyzed in this method, densities of the drop and the fluid it is immersed in cannot be equal. Interfacial surface tension between silicone oil and water is not significantly affected by the addition of NaCl to the water.^14–16^ Therefore, a one molar saltwater solution was used to fill the acrylic container. Pendant drops of low viscosity, high viscosity, and dyed high viscosity oil were grown. The high viscosity and dyed high viscosity oil were grown from the same bottom fill tube used in the original experimental setup. Since the low viscosity oil is more dense than the one molar NaCl solution, an inverted pendent drop was grown from a fill tube that was secured to the mount previously used to lower the glass ring. Images of the pendant drops were analyzed to find the equatorial diameter, and the diameter located one equatorial diameter from the bottom of the drop. Theory from Adamson and Gast^19^ was then applied, resulting in the interfacial tension for each of the cases.

### Velocity measurements and CDF

A Matlab script was written to detect the drop’s shape for each video frame. This data was then used to calculate the overall deformation at each step in the RPM sweep. Additionally, a GUI was written and utilized to hand-track fragments of undissolved dye near the surface of the drop, resulting in azimuthal velocity data. It should be noted that the domains are between 0.1 and 0.5 mm or $\approx$1% of the drop’s diameter and are expected to follow the fluid flow.

The velocity measurements were compared to results from COMSOL simulations, as described in Gulati et al.^9^ Here, we briefly outline the methods. The numerical methods utilized for solving the flow within this geometry are essentially identical to those found in Gulati et al. ^9^. COMSOL finite element method-based modeling was used in solving the flow. The only difference is the polar positioning of the rings; they have been brought in closer to the poles to match the RSD geometry aboard the ISS. A mesh containing quadrilateral elements was created for this geometry. A boundary-layer mesh near the interface was built with a fine distribution of nodes and an increasing refinement near the ring contact points. As in Gulati et al.^9^, a steady-state solver was used to reduce computation times.

### Reporting summary

Further information on research design is available in the Nature Research Reporting Summary linked to this article.

## Supplementary information

Reporting Summary

## Data Availability

The data collected during this study is available from the corresponding authors upon reasonable request.
